# Supplementary material for: Curated and harmonised transcriptomics datasets of interstitial lung diseases
Source: Data Brief. 2025 Oct 14;63:112139. doi: 10.1016/j.dib.2025.112139 (PMC12581653; doi:10.1016/j.dib.2025.112139)
Supplement: Supplementary file 1 [file mmc1.zip › Supplementary_material/RNA-seq/fastQC reports/GSE138283/TRIMMED_SRR10212986_fastqc.html]

TRIMMED\_SRR10212986.fastq.gz FastQC Report 

FastQC Report

Thu 5 Oct 2023  
TRIMMED\_SRR10212986.fastq.gz

## Summary

- Basic Statistics
- Per base sequence quality
- Per sequence quality scores
- Per base sequence content
- Per sequence GC content
- Per base N content
- Sequence Length Distribution
- Sequence Duplication Levels
- Overrepresented sequences
- Adapter Content

## Basic Statistics

| Measure | Value |
| --- | --- |
| Filename | TRIMMED\_SRR10212986.fastq.gz |
| File type | Conventional base calls |
| Encoding | Sanger / Illumina 1.9 |
| Total Sequences | 99082295 |
| Sequences flagged as poor quality | 0 |
| Sequence length | 60-76 |
| %GC | 47 |

## Per base sequence quality

## Per sequence quality scores

## Per base sequence content

## Per sequence GC content

## Per base N content

## Sequence Length Distribution

## Sequence Duplication Levels

## Overrepresented sequences

| Sequence | Count | Percentage | Possible Source |
| --- | --- | --- | --- |
| CCACAAATTATGCAGTCGAGTTTCCCACATTTGGGGAAATCGCAGGGGTCAGCACATCCGGAGTGCAATGGATA | 413887 | 0.4177204413765345 | No Hit |
| CCCACTACCACAAATTATGCAGTCGAGTTTCCCACATTTGGGGAAATCGC | 329671 | 0.3327244287185718 | No Hit |
| CACAAATTATGCAGTCGAGTTTCCCACATTTGGGGAAATCGCAGGGGTCA | 297686 | 0.3004431821043306 | No Hit |
| CCTTAGGCAACCTGGTGGTCCCCCGCTCCCGGGAGGTCACCATATTGATG | 297539 | 0.30029482058323337 | No Hit |
| CCCCACTACCACAAATTATGCAGTCGAGTTTCCCACATTTGGGGAAATCGCAGGGGTCAGCACATCCGGAGTGCA | 293412 | 0.2961295961099811 | No Hit |
| CTTCGATGTCGGCTCTTCCTATCATTGTGAAGCAGAATTCACCAAGCGTT | 243556 | 0.24581182743092497 | No Hit |
| CTGGAGTCTTGGAAGCTTGACTACCCTACGTTCTCCTACAAATGGACCTTGAGAGCTTGTTTGGAGGTTCTAGC | 206691 | 0.20860538202107654 | No Hit |
| CTTAGGCAACCTGGTGGTCCCCCGCTCCCGGGAGGTCACCATATTGATGC | 181567 | 0.18324868232008554 | No Hit |
| CTCCGTTTCCGACCTGGGCCGGTTCACCCCTCCTTAGGCAACCTGGTGGT | 160501 | 0.1619875680110155 | No Hit |
| CACTACCACAAATTATGCAGTCGAGTTTCCCACATTTGGGGAAATCGCAG | 152815 | 0.1542303799079341 | No Hit |
| GGGAGTTTGACTGGGGCGGTACACCTGTCAAACGGTAACGCAGGTGTCCT | 151437 | 0.15283961680540403 | No Hit |
| CAGGCTGGAGTGCAGTGGCTATTCACAGGCGCGATCCCACTACTGATCAG | 151087 | 0.15248637508850596 | No Hit |
| TGATGATGTGTTGTTGCCATGGTAATCCTGCTCAGTACGAGAGGAACCGC | 130133 | 0.1313382981288433 | No Hit |
| CCCCTCCTTAGGCAACCTGGTGGTCCCCCGCTCCCGGGAGGTCACCATAT | 127244 | 0.1284225400713619 | No Hit |
| CCAGGCTGGAGTGCAGTGGCTATTCACAGGCGCGATCCCACTACTGATCA | 123558 | 0.12470240016140119 | No Hit |
| AGCAAATATTCAAACGAGAACTTTGAAGGCCGAAGTGGAGAAGGCTTCCATGTGAACAGCAGTTGAACATGGGTC | 120773 | 0.12189160535694091 | No Hit |
| CAGAAAAGTTACCACAGGGATAACTGGCTTGTGGCGGCCAAGCGTTCATA | 102516 | 0.10346550814148987 | No Hit |
| CTCAGACCGCGTTCTCTCCCTCTCACTCCCCAATACGGAGAGAAGAACGATCATCAATGGCTGACGGCAGTTGC | 99246 | 0.10016522124361371 | No Hit |

## Adapter Content

Produced by FastQC (version 0.11.7)
